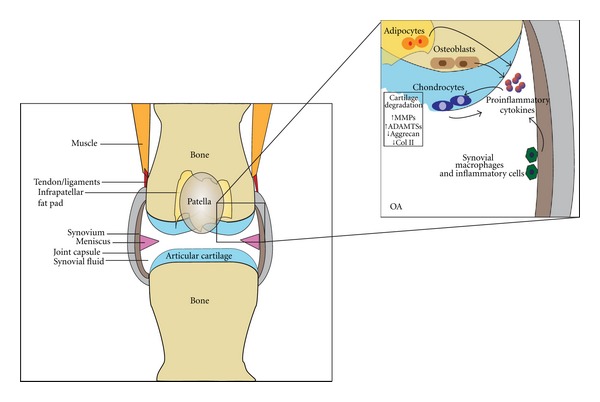# Inflammation and Joint Tissue Interactions in OA: Implications for Potential Therapeutic Approaches

**DOI:** 10.1155/2012/741582

**Published:** 2012-06-18

**Authors:** Roshni Rainbow, Weiping Ren, Li Zeng

**Affiliations:** ^1^Department of Anatomy and Cellular Biology, Tufts University School of Medicine, Boston, MA 02111, USA; ^2^Department of Biomedical Engineering, Wayne State University, Detroit, MI 48201, USA; ^3^Department of Orthopaedic Surgery, Tufts Medical Center, 800 Washington Street, Boston, MA 02111, USA

## Abstract

It is increasingly recognized that the pathogenesis of cartilage degradation in osteoarthritis (OA) is multifactorial and involves the interactions between cartilage and its surrounding tissues. These interactions regulate proinflammatory cytokine-mediated cartilage destruction, contributing to OA progression as well as cartilage repair. This review explores the pathogenesis of OA in the context of the multiple tissue types in the joint and discusses the implications of such complex tissue interaction in the development of anti-inflammatory therapeutics for the treatment of OA.

## 1. Introduction

Situated between bone surfaces, articular cartilage serves as a protective cushion for severe mechanical loading. Extended wear of this tissue can lead to osteoarthritis (OA), a disease estimated to affect over 67 million North Americans by the year 2030 [1]. It has been well studied that OA is a complex, multifactorial inflammatory disease of the whole joint, whose development and progression is significantly mediated by interactions between the joint cartilage and its surrounding tissues. Notably, proinflammatory cytokine-mediated interactions between tissue types contribute to the pathogenesis of OA. The current standard of care for OA involves drug therapies that help to manage and alleviate disease symptoms, with a variety of treatments targeting the inflammatory mediators present during OA pathogenesis. Appropriately, the following review explores OA in the context of how tissues in the joint interact to contribute to inflammation-associated cartilage degradation and the implications of these complex interactions in the development of anti-inflammatory treatments that target the whole joint.

## 2. Proinflammatory Cytokines Mediate Cartilage Degradation

 While the biological onset of OA is not clearly understood, evidence suggests that the progression of cartilage degradation is mediated largely by proinflammatory cytokines, most notably interleukin 1-beta (IL-1*β*) and tumor necrosis factor alpha (TNF*α*) [2, 3]. These cytokines contribute to tissue destruction by disrupting the balance of the catabolic and anabolic activities of chondrocytes, the major cell type of cartilage tissue. Much of the activities of proinflammatory cytokines are mediated by the activation of transcription factor nuclear factor kappa B (NF*κ*B), which further leads to the induction of inducible nitric oxide synthase (iNOS), cyclooxygenase 2 (COX-2), nitric oxide (NO), and prostaglandin E_2_ (PGE-2) [3]. As a result, OA chondrocytes have reduced expression of extracellular matrix components, such as type II collagen (Col II) and aggrecan, as well as increased production of proteolytic enzymes, such as matrix metalloproteinases (MMPs) and peptidases of a disintegrin and metalloproteinase with thrombospondin motifs family (ADAMTSs) [2–5].

## 3. Effect of Joint Tissues in the Pathogenesis of Cartilage Destruction in OA

 While OA has been historically defined as “wear and tear” of articular cartilage, it is increasingly recognized that associated inflammation and subsequent tissue degradation is the result of multiple joint tissue interactions with cartilage. In general, tissues in the vicinity of the joint cartilage consist of subchondral bone, synovium, muscle, tendon, ligament, and fat pad (Figure 1). These tissues regulate proinflammatory cytokine-mediated cartilage destruction, contributing to OA pathogenesis as well as cartilage repair. The following section highlights the important contributions and interactions of the various joint tissues during OA progression.

### 3.1. Bone

 During the progression of OA, subchondral bone undergoes significant morphological changes that include increased bone volume and remodeling, tissue sclerosis, and the formation of osteophytes at the joint margins [6]. These changes correlate with increased signal intensities in MRI images from subchondral bone during OA, which are termed bone marrow lesions (BMLs) [7–9]. Longitudinal studies indicate that alterations in the subchondral bone take place before any detectable radiographic changes in articular cartilage, thereby suggesting that the underlying bone tissue may regulate the initiation of cartilage loss [10]. Correspondingly, during OA, there is an increased expression of inflammatory cytokines in the subchondral bone [11–13]. While the permeability of such bone-derived factors into cartilage tissue is limited in the healthy joint, pathogenesis of OA contributes to cracking within the articular and calcified cartilage zones and promotes diffusion of inflammatory cytokines from the bone into the cartilage through fissures at the osteochondral junction [11]. Indeed, direct signaling between cartilage and bone cells was demonstrated in coculture experiments where OA-derived osteoblasts downregulated expression of proteoglycans and upregulated MMP production in chondrocytes, suggesting an adverse effect of OA subchondral bone toward the overlying cartilage [14–16]. In addition to facilitating diffusion of secreted factors, crevices at the osteochondral junction in late stage OA have also been shown to facilitate the movement of migratory cell clusters that possess strong chondrogenic potential and which may play an important role in cartilage repair [17].

### 3.2. Muscle

 Muscle is long known to provide biomechanical stimuli to cartilage as muscle-generated movement promotes nutrient distribution and maintains homeostasis of chondrocytes [18, 19]. Recently, it was established that muscle tissue also secretes myokines that have anti-inflammatory activities [20]. Coculturing chondrocytes with skeletal muscle cells or muscle cell conditioned medium led to increased cartilage matrix production and increased resistance to proinflammatory cytokine IL-1*β*- and TNF*α*-induced cartilage damage [21, 22]. These results suggest that muscle cells regulate cartilage homeostasis not only through biomechanical forces, but also through biochemical signals. In fact, quadricep muscle weakness, which reduces both biomechanical and biochemical output from muscle, precedes pain and disability of the joint during OA progression, thereby suggesting a possible causative relationship between muscle and cartilage [23].

### 3.3. Adipose Tissue

 It is known that obesity is a major risk factor for the development and progression of OA [24, 25]. While increased weight certainly would contribute to OA through increased mechanical pressure, careful studies indicate that OA incidence in nonweight bearing joints is also higher in obese patients [24, 26], suggesting that adipose tissue has a biochemical output that affects joint cartilage systemically. Fat pads, located within the joint capsules, may also exert a more local effect on cartilage homeostasis, and the infrapatellar fat pad (IFP) was found to increase in size with aging in OA patients [27]. The IFP secretes a substantial amount of adipokines, particularly adiponectin, leptin, and resistin, all which are elevated in the synovial fluid of joints with advanced OA [28–32]. Although other tissues also secrete these factors, the IFP is a major contributor to adipokine presence in the joint. In particular, leptin is considered to be a proinflammatory cytokine and causes catabolic changes in chondrocytes, inducing the expression levels of cartilage degrading enzymes MMPs and ADAMTSs and showing a synergistic role with IL-1*β* to cause cartilage destruction [33–36]. It has been demonstrated that leptin mutant mice, although obese, do not develop OA, thereby strongly suggesting that leptin is required for the development of arthritis in obese mice [37, 38]. Consistent with this study, mutations in the leptin gene are found to be associated with susceptibility to knee OA [39]. However, compared with leptin, the effect of adiponectin in OA still remains controversial. While some reports indicated that adiponectin inhibited IL-1*β*-induced MMP13 induction in primary chondrocytes [40], others showed that it enhanced the production of nitric oxide (NO) and the expression of MMPs in OA chondrocytes [41–43]. It has been shown that serum adiponectin level was significantly lower in OA mice and reversely correlated with OA severity in humans [43, 44]. In contrast, expression levels of adiponectin receptors (AdipoR) were elevated in the articular cartilage of OA patients [41]. Other adipokines such as resistin and visfatin have also been shown to be proinflammatory and play a role in enhancing cartilage degradation by inducing IL-1*β* expression and downregulating proteoglycan synthesis [45]. Taken together, articular cartilage is likely affected by the combinatorial activities of all these adipokines, and it has been found that it is the ratio of adiponectin to leptin in the synovial fluid that predicts pain in knee OA patients [46].

### 3.4. Synovium

 In the synovial joint, articular cartilage is bathed in synovial fluid within the joint cavity [47]. This cavity is lined with two types of cells, synoviocytes that are fibroblast-like cells and macrophages [48]. Synoviocytes secrete lubricin and hyaluronan, two key components involved in the lubrication function of synovial fluid [49]. Synovitis, common in both early- and late-stage OA, is a condition that occurs when the synovium becomes inflicted with inflammation [50]. This condition is marked by dramatically increased secretion of proinflammatory cytokines and proteolytic enzymes from synovium-lining cells [51–55]. Proinflammatory cytokines, most notably IL-1*β*, IL-6, and TNF*α*, are thought to mediate the progression and pain associated with this disease [50, 56]. Adipokines, such as resistin [45], are also increasingly expressed by the synovium during OA, as is osteopontin [57], a cytokine whose increased expression levels have been correlated with disease severity. Furthermore, *ex vivo *coculturing of joint capsule tissue with damaged cartilage has demonstrated the contribution of the synovium to shifting chondrocyte metabolism towards matrix degradation [58–60]. In addition to providing signaling molecules that regulate articular cartilage gene expression, synoviocytes themselves can differentiate into chondrocytes. Recently, a population of CD73-positive cells were identified in the synovium, which possess mesenchymal stem cell-like characteristics, such as slow-cycling and the ability to differentiate into multiple lineages, including chondrogenic and osteogenic, *in vitro* [61, 62]. Thus, these cells may serve as an attractive cell source for cartilage regeneration and repair [63]. Indeed, synoviocytes were proposed to contribute to the layer of fibrous tissue, called the “pannus-like” layer, which is frequently found to be overlying the articular cartilage in OA joints [64, 65]. However, a recent study indicates that synoviocytes from OA patients do not have the ability to colonize adjacent cartilage [66], suggesting that this tissue may not be derived directly from synoviocytes. In the same study, it was shown that synoviocytes from rheumatoid arthritis (RA) patients, on the other hand, had the ability to attach to adjacent cartilage and to even spread to other joints of the body [66]. This indicates a distinct difference between synoviocytes of OA and RA patients, suggesting that the joint capsule is permeable enough to allow not only fluid or secreted factors, but also cells to pass through. 

## 4. Therapeutic Approaches for OA

 The current standard of care for OA involves the use of drugs that help to manage and alleviate disease symptoms. Common early-stage OA treatment options include the use of analgesics and nonsteroidal anti-inflammatory drugs (NSAIDs) [67, 68]. While both analgesics and NSAIDs are utilized to alleviate pain symptoms, NSAIDs specifically act by targeting the inflammation associated with OA through the inhibition of COX [67]. The COX enzyme mediates the synthesis of prostaglandins (PG), biomolecules involved in inflammation, and two classes of COX inhibitors exist, COX-1 and COX-2, with COX-2 inhibitors being developed to avoid the gastrointestinal side effects associated with the long-term use of COX-1 inhibitors. Both analgesics and NSAIDs can be administered orally or topically; however, the latter has proven to be a less effective [69, 70]. Despite their popularity, NSAIDs do not slow down the progression of OA through any disease modification and the efficacy of treatment as compared to placebo therapy is often times minimal [71]. Additionally, there is controversy as to whether NSAIDs actually inhibit cartilage degradation or worsen the conditions of OA by providing an analgesic effect or adverse side effects [68].

 An alternative treatment option to NSAIDs includes the use of hyaluronic acid (HA) and corticosteroids [72]. While endogenous HA provides adequate viscoelastic and lubricating properties to maintain joint homeostasis in a healthy joint, during OA, the properties of HA are diminished and contribute to further cartilage destruction [73, 74]. Intra-articular injection of HA has been shown to inhibit cartilage degradation, induce matrix synthesis, reduce pain symptoms, and downregulate the expression of proinflammatory mediators [74, 75]. It is important to note that the effect of HA is dependent on its molecular weight (MW), as only cross-linked or higher MW HA is effective in mitigating inflammation, while lower MW HA or HA fragments are proinflammatory [76, 77]. Indeed, a recent report of a clinical trial using HA therapy indicated that intermediate MW HA was more superior as compared to low MW HA in alleviating knee OA symptoms [78]. Similar to HA, intra-articular administration of low dose corticosteroids has shown to reduce both the expression of proinflammatory mediators and the permeability in the inflamed area by lessening vascular dilation [79], as well as decrease inflammation and swelling in OA joints, thereby managing pain and enhancing joint mobility [80]. For both HA and corticosteroid treatment, rates of adverse side effects are low; however, it is worth noting that corticosteroids, particularly at a higher level, may have a damaging effect toward bone formation [69, 81]. As OA is a disease of the whole joint, it is especially important to consider the effect of these drugs on all cell types in the joint. Furthermore, success with such therapies has been shown to be beneficial only the first few weeks after intra-articular injection. As a result, repeated and invasive injection treatments are needed for maintaining long-term efficacy [67].

 Antibiotics, such as doxycycline, have also been explored for their role as disease modifying drugs in the treatment of OA. Preclinical trials using a guinea pig OA model and magnetic resonance imaging (MRI) suggest the protective role of this drug in lessening cartilage volume loss and inhibiting MMP activity [82]. However, this treatment option is controversial as the definitive role of doxycycline during OA treatment is not well understood. For example, an OA rabbit model has demonstrated that while doxycycline treatment was inconclusive in definite treatment of OA, cartilaginous changes observed in these studies suggest a potential role of doxycycline in disease modification [83]. Human trials exploring doxycycline as a treatment option for knee joint OA suggest that while this drug may slow down the rate of joint space narrowing, there lacks definitive evidence of symptomatic improvement [84]. Furthermore, *in vitro* studies on the effect of doxycycline on cartilage degradation have shown that while this antibiotic has an inhibitory role on aggrecanase expression, there is no indication of proteoglycan synthesis or loss under inflammatory conditions [85].

 As proinflammatory cytokines play a significant role in mediating cartilage degradation and OA-associated inflammation, the inhibition of such factors, particularly IL-1*β* and TNF*α*, could be a viable therapy for slowing down the progression of OA. In accordance, new classes of drugs, called disease modification OA drugs (DMOADs), have become increasingly promising therapies [71, 86]. While still an emerging therapy, DMOADs aim to provide structural and metabolic changes to the inflammatory joint environment, with hopes to slow down the progression of OA and possibly provide healing to already damaged cartilage. Drugs, such as inhibitors of iNOS and avocado-soyabean unsaponifiables, are currently being explored in trial for their role in reducing OA-associated inflammation. While iNOS inhibitors curb the activation of MMPs, avocado-soyabean unsaponifiables have an anti-inflammatory effect on chondrocytes and inhibit the breakdown of cartilage matrix and are currently being explored in Phase III clinical trails [71]. Other drugs, such as statins, commonly prescribed for cholesterol reduction, have shown promising results *in vitro* to reduce MMP-3 production in IL-1*β* stimulated chondrocytes [87].

 While DMOADs are being explored as an alternative to current treatment options, success of these therapies will require further study. For example, IL-1*β* receptor antagonists have been shown to have a promising effect in the inhibition of structural changes occurring during OA in rabbit, canine, and horse models [2], but remain inconclusive in humans [68]. Caspase-1 inhibitors have been shown to reduce joint damage mediated by IL-1 in murine models; however, adverse side effects in humans have resulted in clinical trials being put on hold [67].

 As highlighted in this paper, multiple tissues contribute to the inflammatory microenvironment and subsequent cartilage degradation during OA pathogenesis, and these tissue interactions may provide an attractive disease modification pathway with respect to therapeutic treatment. For example, subchondral bone, with its contributions to cartilage degradation during OA, is one tissue type that is currently being explored as a disease modification target for three DMOADs in clinical trial. Currently in Phase III clinical trial [71], calcitonin, a hormone regulating calcium homeostasis, has been shown to inhibit MMP activity and subsequent cartilage degradation [88]. Additionally, bone morphogenetic protein 7 (BMP-7), a potent bone-inducing agent, has been shown to stimulate proteoglycan, collagen, and HA synthesis in cartilage [89, 90], while vitamin D has been shown to reduce the progression of osteoarthritis [91]. Both BMP-7 and vitamin D are in Phase II trials [71].

 Because OA joints have decreased mineral content and increased bone turnover, bisphosphonates have been also explored as potential DMOADs with respect to targeting the subchondral bone during OA progression [92]. These molecules have been shown to inhibit bone resorption and reduce the synthesis of inflammatory mediators as well as increase cartilage volume in canine OA models [93, 94]. The combined use of bisphosphonates with NSAIDs as a therapy for early OA has shown to preserve bone mass, decrease osteophyte formation, and increase OA severity [95].

 Modification of the contributions of the synovial membrane during OA has also been explored as a treatment option through the use of chondroitin sulfate, which has been shown to reduce signs of synovitis and inflammation in the joint space [96]. Likewise, contributions of adipose tissue to OA are also being explored as a potential therapeutic target.

## 5. Advancements in Drug Delivery

 When present, OA often affects multiple joints, and as a result, a drug therapy that can target a variety of joint tissues in the body is highly desirable. For example, as discussed above, many NSAIDS and COX inhibitors can be administered orally to have a systemic affect to the whole body. While systemic delivery allows the treatment of multiple affected joints, localized drug delivery to the joint microenvironment is an optimal therapeutic approach when the number of joints affected is limited. Here, oral or injection delivery may not provide adequate drug concentrations or release kinetics. Furthermore, high systemic concentrations that are required to achieve the appropriate therapeutic concentrations within the joint space may result in adverse side effects [97–99]. In the intra-articular space, for example, synovial membrane permeability can result in increased diffusion of drugs out of the joint space, and together with shorter drug half-life, can result in shorter joint residence times. Here, while injections are a possible method to increase local delivery to the joint space, they are quite invasive and may be increased in frequency to achieve efficacy. To circumvent such issues, there is interest in developing localizable methods that can achieve sustained, pro-longed delivery, particularly for the intra-articular joint space [100]. Acting as a depot for the therapeutic drug, liposome- and polymeric-based systems have been widely explored* in vivo* for the controlled delivery of OA treatment drugs [101]. Here, the therapeutic drug is entrapped within a liposome or a biodegradable polymeric matrix, allowing for prolonged bioavailability and increasing drug residence time in the joint cavity. The stability and the degradation of the lipids and polymers composing such structures allow for control over the timing and dosage of delivery [101, 102]. While such methods are a promising delivery strategy for intra-articular delivery, only one clinical therapy exists to date for OA that combines the use of intra-articular injections with liposomes containing dexamethasone-21-palmitate [98]. With respect to polymeric-based intra-articular drug treatments, albumin microspheres have been explored for delivery of NSAIDS including diclofenac sodium [103] and COX-2 inhibitor celecoxib [104]. Likewise, poly(lactic acid) and poly(lactic-coglycolic acid) (PLA/PLGA) have been explored as delivery matrices for NSAIDs, such as betamethasone sodium phosphate [105], corticosteroids, such as methylprednisolone [106], and DMOADs such as BMP-7 [107].

## 6. Conclusion

 OA is recognized as a multifactorial inflammatory disease of the whole joint, with a complex pathomechanism involving interactions between the multiple joint tissues. Furthermore, the development of OA is largely mediated by proinflammatory cytokines and their subsequent contributions to cartilage degradation. Current therapies manage OA largely by alleviating symptoms and pain; however, drugs that intercede the inflammatory OA pathway are actively being explored as therapeutic options. Such drugs may be delivered systemically, which may be particularly relevant for the treatment of multiple joints within the body at once, or may be delivered locally to a single joint space using intra-articular injections or other localizable drug delivery methods. Regardless of the delivery modality, a varying collection of approaches to OA will likely be required to exploit the complex interactions between joint tissues.

## Figures and Tables

**Figure 1:**